# More new records of spider wasps from Colombia (Hymenoptera, Pompilidae)

**DOI:** 10.3897/zookeys.658.10538

**Published:** 2017-02-23

**Authors:** Cecilia Waichert, Fernando Fernández, Valentina Castro-Huertas, Juanita Rodriguez, James P. Pitts

**Affiliations:** 1 Universidade Federal do Espírito Santo, Departamento de Ciências Biológicas, Vitória, ES, Brasil; 2 Instituto de Ciencias Naturales, Universidad Nacional de Colombia, Bogotá D.C., Colombia; 3 Laboratório de Entomologia Sistemática, Instituto de Biociências, Universidade Federal do Rio Grande do Sul, Porto Alegre, RS, Brasil; 4 Australian National Insect Collection, CSIRO, Canberra, ACT, Australia; 5 Department of Biological Sciences, Auburn University, Auburn, AL, USA; 6 Department of Biology, Utah State University, Logan, UT, USA

**Keywords:** New combination, Pepsinae, Pompilinae

## Abstract

*Aporinellus* Banks, *Austrochares* Banks and *Dicranoplius* Haupt are new generic records for Colombia, as well as the species *Dipogon ariel* Banks, *Evagetes peruana* Banks, and *Euplaniceps notabilis* (Smith). Five new combinations are formally endorsed: *Aimatocare argentinica* (Banks), **comb. n.**; *Aimatocare longula* (Banks), **comb. n.**; *Aimatocare imitator* (Evans), **comb. n.**; *Aimatocare impensa* (Evans), **comb. n.**; *Aimatocare vitrea* (Fox), **comb. n.** Although these names have been used in Pompilidae, no formal nomenclatural act had been proposed. The presence of *Chirodamus paramicola* Roig-Alsina, previously reported with uncertainty, is confirmed. Finally, a new combination for *Euplaniceps notabilis* (Smith), **comb. n.** is proposed based on molecular phylogenetics and morphological data. The Colombian fauna of Pompilidae sums up to 38 genera and approximately 150 species.

## Introduction

The mostly solitary spider wasps (Hymenoptera: Pompilidae) are a widespread group of more than 1,000 species in four subfamilies and 60 genera in the Neotropics (Fernández 2000, Hanson and Wasbauer 2006). Despite some recent progress in the taxonomic study of Neotropical pompilids (*e. g.* Rodriguez et al. 2010, Waichert et al. 2012, Waichert et al. 2015), their nomenclature and systematics remains incipient. As a result, several genera lack identification keys and urge taxonomic revision, and many species remain to be described.

With the growing anthropogenic pressures on the Neotropical forests, one of the main concerns is the loss of biodiversity, which increases the need of prioritizing taxonomic studies in these areas. The authors are currently engaged in the monograph of spider wasps from Colombia, including keys to subfamilies, tribes, genera and species of several genera. This endeavor favored the publication of new genera and species records (Castro-Huertas et al. 2014). Because we are approaching the completion of the book’s manuscript, it is desirable to publish some additional new records of genera and species, as well as relevant nomenclatural acts, as a separate note from the forthcoming book.

To date, the fauna of spider wasps from Colombia has 150 known species, belonging to 38 genera and four subfamilies.

## Methods

*Examined specimens.* The specimens are deposited in the Entomological collection at Instituto Alexander von Humboldt, Villa de Leiva, Colombia (**IAvH-E**), Instituto de Ciencias Naturales, Universidad Nacional de Colombia, Bogotá, Colombia (**ICN**), Entomological Collection of the Museo Javeriano de Historia Natural, Pontificia Universidad Javeriana, Bogotá, Colombia (**MPUJ**) and Museo Entomológico Universidad del Valle, Cali, Colombia (**MUSENUV**). *Dicranoplius* Haupt specimens from the Utah State University Entomology Collection, Logan, UT (**EMUS**) were examined.

## Results

The following species and genera are reported for the first time in Colombia.

### Subfamily Pompilinae

#### *Aporinellus* Banks

##### Aporinellus aff. medianus



Banks, 1917

###### Specimen data.

**Magdalena.** 1♂, PNN Tayrona, Palangana [11°20'0"N, 74°2'0"W]; 30 m, malaise, 4–23 May 2001, R. Henríquez, M1765 (IAvH-E); 1♂, Neguanje, [11°20'0"N, 74°2'0"W], 10 m, Malaise, 28 Jul - 18 Aug 2001, R. Henríquez, M2019 (IAvH-E).

###### Comments

This is the first record of the genus for Colombia and northern South America. The known distribution of *Aporinellus* is worldwide, except Australia (Evans 1966). The studied specimens probably belong to an undescribed species that will be studied elsewhere.

### *Austrochares* Banks

#### Austrochares aff. mexicanus



Dreisbach, 1966

##### Specimen data.

**Magdalena.** 1♀, PNN Tayrona, Neguanje, [11°20'N, 74°2'W]; 10 m, malaise, 20 Apr–4 May 2001, R. Henríquez, (IAvH-E), 1♂, 4–23 May 2001, R. Henríquez, (IAvH-E).

##### Comments.

This is the first record of the genus for Colombia and northern South America. *Austrochares* was previously known from Mexico to Argentina, including Brazil, Chile, and Peru (Evans 1969). The studied specimen keys out to *Austrochares mexicanus* in Evans’s (1969) key, but it probably belongs to an undescribed species that will be further studied elsewhere.

### *Dicranopilus* Haupt

#### Dicranopilus aff. areatus



(Taschenberg, 1869)

##### Specimen data.

**Bolívar.** 2♂, Santuario de Fauna y Flora Los Colorados, Alto el Mirador, 6–24 Oct 2001, E. Deulufeut (IAvH-E). **Magdalena.** 8♂, 1♀, *PNN Ta*y*rona*, Neguanje, 20 Apr–4 May 2001; 9♂, 2♀, 4 –23 May 2001; 3♂, 14–28 Jul 2001; 11♂, 28 Jul–18 Aug 2001; 12♂, 17–27 Sept 2001, R. Henriquez (EMUS, IAvH-E).

##### Comments.

This is the first record of the genus for Colombia. *Dicranoplius* is restricted to the Neotropics, to both temperate and tropical South America. It was previously known from Trinidad south to Argentina (Evans 1969). The studied specimens key out as *Dicranoplius areatus* in Evans’s (1969) key, but they belong to an undescribed species, which will be described elsewhere.

### *Euplaniceps* Haupt

#### Euplaniceps notabilis



(Smith, 1860) comb. n.

Aporus (Aporus) notabilis notabilis (Smith, 1860), Mem. Am. Entomol. 20: 52.
Planiceps notabilis Smith, 1860, Jour. Ent., 1:80 [Type: female, Mexico] – Cresson, 1867, Trans. Amer. Ent. Soc., 1: 137.
Pompilus (Planiceps) notabilis Cameron, 1893, Biol. Centr.-Amer., Hymen. II, p. 186.
Pompilus flavomarginatus Cameron, 1893, ibid, p. 191 [Type: female, Mexico: Yucatan. BMNH no. 19,703] Syn. by Evans 1966.
Odontaporus notabilis Bradley, 1944, Trans. Amer. Ent. Soc., 70: 114–115.

##### Specimen data.

See Rodriguez et al. (2015), Appendix S1.

##### Comments.

This is the first record of this species for Colombia. *Euplaniceps notabilis* was previously recorded from Mexico to Costa Rica (Evans 1966). The new combination is proposed based on molecular phylogenetic analyses (Rodriguez et al. 2015), which included *Aporus notabilis* (labeled “*Euplaniceps* sp.”, voucher PO484) in the *Euplaniceps* clade, sister to the Antillean *Drepanaporus*. This species had previously been included in the genus *Odontaporus* by Bradley (1944) based on the presence of a tooth in the margin of the inferior mandible and bare eyes. Bradley (1944) did not mention the presence of this tooth in many *Euplaniceps* species (see Colomo de Correa 1998). Evans (1966) included this species in *Aporus* based mainly on the second submarginal cell receiving only one recurrent vein. This character is very variable even within *Euplaniceps* species (JR pers. obs.), where the second submarginal cell can receive one or two recurrent veins, with the second recurrent vein sometimes being interstitial with the second intercubital vein. Moreover, females of *Aporus notabilis* do not have a v-shaped spatium frontale as all other *Aporus*, but a flattened area between the antennal alveoli on the same plane as the clypeus which is found in many *Euplaniceps* species (see Colomo de Correa 1998). Finally, the male genitalia have parameres with parallel-side edges and truncated apex, which is a diagnostic character of *Euplaniceps* males. The two subspecies, *Aporus notabilis notabilis* and *Aporus notabilis pulchritarsis*, are herein included in *Euplaniceps*.

### *Evagetes* Lepeletier

#### Evagetes peruana



(Banks, 1947)

##### Specimen data.

**Boyacá.** 22♂, SFF Iguaque, Cabaña Chaina, [5°25'N, 73°27'W], 2,600 m, malaise, 9–26 Sept 2002, A. Roberto (IAvH-E).

##### Comments.

This is the first record of this species for Colombia and east of the Andes. *Evagetes* is most diverse in the Holarctic region, having South American species occurring west of the Andes (Argentina, Chile and Peru) (Evans 1966; Fernández 2000). This species was previously known from Trujillo, Peru (Banks 1947).

### *Priochilus* Banks

#### Priochilus formosum hondurense



Dreisbach, 1950

##### Specimen data.

**Vichada**. 1♀, Centro Gaviotas, 170 m, 17 Oct 1989, F. Fernández leg. (ICN).

##### Comments.

Fernández (2000) cited the occurrence of *Priochilus formosum hondurense* Dreisbrach, 1950 in Colombia (Orinoco region), without providing specimen data and/or specimen voucher. We add a female specimen to this species’ range. *Priochilus formosum hondurense* is known from females only and had been known from Honduras to Costa Rica (Evans 1966).

#### Priochilus imperius



Banks, 1944

##### Specimen data.

**Meta.** 1♀, RNN La Macarena, Caño La Curía, 580 m., 25 Dec 1986, F. Fernández leg. (ICN).

##### Comments.

Fernández (2000) reported the species from Bolivia, Brazil, Ecuador, Colombia (Meta) and in Peru (*apud* Evans 1966). Additionally, the Catalogue of Life (www.catalogueoflife.org) has added Chile to *Priochilus imperius* list of occurrences. Specimen data and/or specimen voucher are not provided by Fernández (2000), making record questionable. Here we record a female specimen from Colombia. *Priochilus imperius* was described based on specimens from Bolivia, British Guiana, Ecuador, Peru, and Surinam (Banks 1944).

### Subfamily Pepsinae

#### Aimatocare



Roig-Alsina

##### Note.

Roig-Alsina (1989) established the genus *Aimatocare* for the *Chirodamus argentinicus* species-group, as delimited by Evans (1968), but did not propose new combinations for the newly established genus. Herein, we formalize the following nomenclatural acts: *Aimatocare argentinica* (Banks), **comb. n**.; *Aimatocare imitator* (Evans), **comb. n**.; *Aimatocare longula* (Banks, 1946), **comb. n**.; *Aimatocare impensa* (Evans), **comb. n**.; and *Aimatocare vitrea* (Fox), **comb. n**.

#### Aimatocare longula



(Banks, 1946)

##### Specimen data.

**Cauca.** 1♀, Morales, El Pomarroso, Finca El Recuerdo, [2°48'34.5"N, 76°37'15.8"W], 1,600 m, by hand, 12 Sept 2013, D. Caraball (MUSENUV).

##### Comments.

This is the first record of this species for Colombia. It was previously known from Bolivia, Brazil and Peru (Fernández 2000).

### *Chirodamus* Haliday

#### Chirodamus paramicola



Roig-Alsina, 1984

##### Specimen data.

**Cundinamarca.** 1♀, PNN Chingaza, 3,400 m, hand collection, Nov 1988, G.D. Amat (MPUJ).

##### Comments.

Roig-Alsina (1984) recognized five species mostly from southern South America, with one species, *Chirodamus paramicola*, in Venezuela and Colombia. The holotype and paratypes are from the Venezuelan Andes, and the single specimen from Colombia was collected in Nariño (southwestern Colombia) and deposited in London without further study. We found a new specimen from Chingaza Park, which slightly differs from the type series by having different fore leg coloration. The Colombian specimen studied here comprises a possible geographical and morphological bridge between the Merida (Venezuela) and Nariño specimens.

### *Dipogon* Fox

#### Dipogon ariel



Banks, 1946

##### Specimen data.

**Boyacá.** 1♀, SFF Iguaque, Cabaña Chaina, [5°25'0"N, 73°7'0"W], 2,600m, malaise, 10 Jun 2001, 28 Jun 2001, P. Reina (IAvH-E).

##### Comments.

This is the first record of this species for Colombia. *Dipogon ariel* was previously known from Ecuador (Banks 1946).

## Supplementary Material

XML Treatment for Aporinellus aff. medianus

XML Treatment for Austrochares aff. mexicanus

XML Treatment for Dicranopilus aff. areatus

XML Treatment for Euplaniceps notabilis

XML Treatment for Evagetes peruana

XML Treatment for Priochilus formosum hondurense

XML Treatment for Priochilus imperius

XML Treatment for Aimatocare

XML Treatment for Aimatocare longula

XML Treatment for Chirodamus paramicola

XML Treatment for Dipogon ariel
